# Complete mitochondrial genomes of June sucker and Utah sucker (*Chasmistes liorus* and *Catostomus ardens*)

**DOI:** 10.1080/23802359.2022.2055984

**Published:** 2022-03-28

**Authors:** Peter C. Searle, Jackson B. Linde, Jillian R. Campbell, Andrea L. Kokkonen, Dennis K. Shiozawa, Mark C. Belk, R. Paul Evans

**Affiliations:** a Department of Biology, Brigham Young University, Provo, UT, USA; b Department of Microbiology and Molecular Biology, Brigham Young University, Provo, UT, USA; c Monte L. Bean Life Science Museum, Brigham Young University, Provo, UT, USA

**Keywords:** *Chasmistes liorus*, *Catostomus ardens*, Catostomidae, sucker

## Abstract

The relationship between June sucker (*Chasmistes liorus*, Jordan, 1878) and Utah sucker (*Catostomus ardens*, Jordan & Gilbert, 1881) has been a matter of controversy since the mid 1900s. *Chasmistes liorus* is endemic to Utah Lake, UT and has a subterminal mouth adapted for pelagic feeding. *Catostomus ardens* is widely distributed throughout the Bonneville Basin and Upper Snake River Basin and has a ventral mouth adapted for benthic feeding. *Chasmistes* has been recognized as a separate ancient genus. Despite being morphologically distinct, no study has successfully identified residual genetic markers that separate these species. Of these studies, several have used a subset of mitochondrial genes, but no study has analyzed the complete mitochondrial genomes (mitogenomes) of these suckers (Pisces: Catostomidae). To further explore the genetic relationships between these species, we report the complete mitogenomes of *Chasmistes liorus* and *Catostomus ardens*. DNA was sequenced using an Illumina HiSeq 2500 system and mitogenomes were assembled and annotated using Geneious v. 2021.2 and MitoAnnotator, respectively. The mitogenomes of *Chasmistes liorus* and *Catostomus ardens* are both 16,623 bp and are ∼0.072% divergent. We examine the phylogenetic relationship between *Chasmistes liorus* and *Catostomus ardens* using 33 mitogenomes, representing 16 species, from Catostomidae. Our data suggest that *Chasmistes liorus* is sister to *Catostomus ardens*. Additional samples from multiple localities and/or cohorts of these species will allow us to better resolve the complicated phylogenetic relationships between these species.

Catostomidae – commonly known as ‘suckers’ – is a diverse family of freshwater fish (76 species) found in North America and Asia (Bagley et al. 2018). June sucker (*Chasmistes liorus*, Jordan, 1878), is a member of *Chasmistes*, an ancient genus (four species) of fish endemic to plankton-rich, terminal lakes in western North America. *Chasmistes* have subterminal mouths adapted for pelagic feeding. Utah sucker (*Catostomus ardens*, Jordan & Gilbert, 1881), is a member of *Catostomus*, a genus (26 species) that has ventral mouths adapted for benthic feeding (Cole et al. 2008). June sucker is endemic to Utah Lake, while Utah sucker is widely distributed throughout the Bonneville Basin and Upper Snake River basin.

The relationship between June sucker and Utah sucker has been a matter of controversy since the mid-1900s, largely due to the presence of suckers with intermediate mouth morphologies in Utah Lake (Miller and Smith 1981). Initially, it was hypothesized that *Chasmistes* evolved 7–10 mya and that the presence of these ‘intermediate’ suckers was the result of introgression between the ancient lineages of *Chamistes* and *Catostomus* (Smith et al. 2018). However, no study has successfully identified residual genetic markers that separate these species (Mock et al. 2006; Cole et al. 2008). An alternative hypothesis is that the *Catostomus ardens* in Utah Lake differentiated into two forms: a planktivorous form (*Chasmistes liorus*) and benthivorous form (*Catostomus* ardens). This hypothesis suggests that the presence of intermediates could represent a convergence toward, or divergence away from a June sucker-like morphology, dependent on environmental conditions (Cole et al. 2008).

Several studies have used various mtDNA genes to explore the genetic relationships between June sucker and Utah sucker. Although proponents of both hypotheses agree that mtDNA between June sucker and Utah sucker is similar, they disagree as to whether the small divergence in mtDNA is (1) evidence of a recent hybridization event followed by asymmetric replacement of *Chasmistes* mtDNA or (2) evidence of incipient speciation with only a few differences due to limited time of divergence (Smith et al. 2018). We report the complete mitochondrial genomes of June and Utah sucker to further explore the mtDNA-based relationships between these species.

We collected larval June sucker (brood stock population) and Utah sucker (introduced population in Strawberry Reservoir, Utah) in 2014 from the State of Utah’s Fisheries Experiment Station (Logan, UT, USA; 41.7363, −111.8695). We euthanized specimens with tricaine methanesulfonate (MS-222, MilliporeSigma, St. Louis, MO, USA) and then stored the specimens in RNAlater (MilliporeSigma, St. Louis, MO, USA) at −20 °C (Utah Division of Wildlife Resources’ scientific collection permit #1COLL5950, IACUC-approved protocol #15-0602). We extracted DNA using the whole specimen and sequenced our samples using an Illumina HiSeq 2500 system (Illumina, San Diego California, USA) at Brigham Young University’s DNA Sequencing Center (Provo, Utah, USA). The DNA samples were deposited in the Life Science Museum, Brigham Young University (curator of fishes: Jerald B. Johnson, jerry.johnson@byu.edu) under accession numbers 318670 and 318683.

We checked sequence quality using FastQC (Andrews 2010), assembled sequences with Geneious v. 2021.2 (Biomatters Ltd., Auckland, New Zealand) and annotated mitogenomes using MitoAnnotator (Iwasaki et al. 2013). Mitogenomes were assembled using *Catostomus catostomus* (MG570441) as a reference. We acquired 31 mitogenomes (14 species) representing all available mitogenomes for Catostomidae from GenBank. We generated multiple sequence alignments separately for each of the 13 protein-coding genes using MAFFT v. 7.475 (Katoh et al. 2002) with Geneious v. 2021.2 (Biomatters Ltd., Auckland, New Zealand) before concatenating them into a final alignment that was 11,441 bp. A phylogenetic tree, inferred by maximum likelihood, was constructed using IQ-Tree (Trifinopoulos et al. 2016) on the CIBIV, Austria webserver (available at http://iqtree.cibiv.univie.ac.at). Our IQ-tree run used ModelFinder (Kalyaanamoorthy et al. 2017) to find the best-fit model according to the Bayesian Information Criterion as GTR + F + I + G4. Additional parameters included 10,000 ultrafast bootstraps, 1,000,000 maximum iterations and 1,000 SH-aLRT replicates.

The complete mitogenomes of *Chasmistes liorus* (MZ907583) and *Catostomus ardens* (MZ907582) were both 16,623 bp, containing 13 protein coding genes, 22 tRNAs, two rRNAs, and the control region. Consistent with previous studies (Mock et al. 2006; Smith et al. 2018), the mtDNA of *Chasmistes liorus* and *Catostomus ardens* were very similar. Their complete mitogenomes were ∼0.072% divergent, with all differences occurring in the protein-coding genes. In comparison, the complete mitogenomes of the putative sister taxa *Catostomus latipinnis* and *Xyrauchen texanus* were 4.394% divergent.

In our within-taxon-limited phylogeny, *Chasmistes liorus* is sister to *Catostomus ardens* (Figure 1). This is not surprising as there are only 12 nucleotide differences in their entire mitogenomes. However, without additional representatives of *Chasmistes* and *Catostomus*, we cannot reject either the incipient speciation or the asymmetric hybridization hypotheses. In addition, the placement of other members of *Catostomus* is consistent with previous studies that failed to resolve *Catostomus* as monophyletic (Bagley et al. 2018).

**Figure 1.**
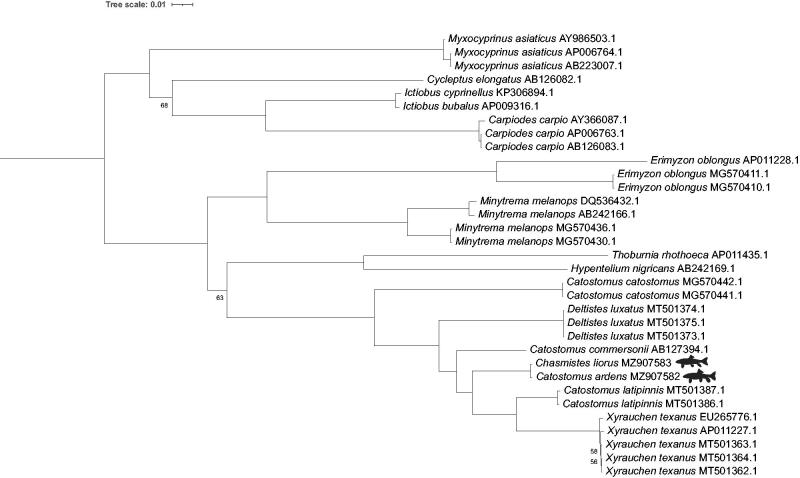
Phylogenetic tree inferred by maximum likelihood using IQ-Tree from 33 Catostomidae mitogenomes, representing 16 species*. Cobitis striata* (AB054125), *Cyprinus carpio* (MK088487) and *Gyrinocheilus aymonieri* (AB242164) were used as outgroups but are not displayed. Bootstrap values >95 are not shown. Reproduction of June sucker and Utah sucker silhouettes was done with permission from © Joseph R. Tomelleri.

## Data Availability

The GenBank, BioProject, Bio-Sample, and SRA accession numbers for *Chasmistes liorus* are MZ907583, PRJNA757012, SAMN20938802, and SRR15598911. The GenBank, BioProject, Bio-Sample, and SRA accession numbers for *Catostomus ardens* are MZ907582, PRJNA757012, SAMN20938803, and SRR15598910. All data is available through NCBI at https://www.ncbi.nlm.nih.gov/.
